# Development and external validation of a radiomics model for assessment of HER2 positivity in men and women presenting with gastric cancer

**DOI:** 10.1186/s13244-022-01361-x

**Published:** 2023-02-01

**Authors:** Huiping Zhao, Pan Liang, Liuliang Yong, Ming Cheng, Yan Zhang, Minggang Huang, Jianbo Gao

**Affiliations:** 1 Department of CT, Shaanxi Provincial People’s Hospital, No. 256, Youyi West Road, Xi’an, 710068 Shaanxi Province China; 2 Department of Radiology, The First Affiliated Hospital of Zhengzhou University, No. 1, East Jianshe Road, Zhengzhou, 450052 Henan Province China; 3 Henan Key Laboratory of Image Diagnosis and Treatment for Digestive System Tumor & Henan International Joint Laboratory of Medical Imaging & Henan Engineering Laboratory of Tumor Imaging & Henan Key Laboratory of CT Imaging & Zhengzhou Key Laboratory of Medical Imaging Technology and Diagnosis, No. 1, East Jianshe Road, Zhengzhou, 450052 Henan Province China; 4 Department of Medical Information, The First Affiliated Hospital of Zhengzhou University, No. 1, East Jianshe Road, Zhengzhou, 450052 Henan Province China

**Keywords:** Computed tomography, Radiomics, Nomogram, HER2 testing, Gastric cancer

## Abstract

**Background:**

To develop and externally validate a conventional CT-based radiomics model for identifying HER2-positive status in gastric cancer (GC).

**Methods:**

950 GC patients who underwent pretreatment CT were retrospectively enrolled and assigned into a training cohort (*n* = 388, conventional CT), an internal validation cohort (*n* = 325, conventional CT) and an external validation cohort (*n* = 237, dual-energy CT, DECT). Radiomics features were extracted from venous phase images to construct the “Radscore”. On the basis of univariate and multivariate analyses, a conventional CT-based radiomics model was built in the training cohort, combining significant clinical-laboratory characteristics and Radscore. The model was assessed and validated regarding its diagnostic effectiveness and clinical practicability using AUC and decision curve analysis, respectively.

**Results:**

Location, clinical TNM staging, CEA, CA199, and Radscore were independent predictors of HER2 status (all *p* < 0.05). Integrating these five indicators, the proposed model exerted a favorable diagnostic performance with AUCs of 0.732 (95%CI 0.683–0.781), 0.703 (95%CI 0.624–0.783), and 0.711 (95%CI 0.625–0.798) observed for the training, internal validation, and external validation cohorts, respectively. Meanwhile, the model would offer more net benefits than the default simple schemes and its performance was not affected by the age, gender, location, immunohistochemistry results, and type of tissue for confirmation (all *p* > 0.05).

**Conclusions:**

The conventional CT-based radiomics model had a good diagnostic performance of HER2 positivity in GC and the potential to generalize to DECT, which is beneficial to simplify clinical workflow and help clinicians initially identify potential candidates who might benefit from HER2-targeted therapy.

**Supplementary Information:**

The online version contains supplementary material available at 10.1186/s13244-022-01361-x.

## Key points

- Radiomics model holds promise in identifying HER2 positivity in GCs.
- The conventional CT-based HER2-specific radiomics model might generalize to DECT.
- This study provides an imaging surrogate for stratifying GC by HER2 expression.

**Supplementary Information:**

The online version contains supplementary material available at 10.1186/s13244-022-01361-x.

## Background

Gastric cancer (GC) remains a globally important disease, with high incidence and mortality rates in men and women [1, 2]. As a unique subtype of GC, HER2 overexpression-positive (HER2-p) adenocarcinoma displays specific clinical features [3], and its treatment modalities are different from HER2 overexpression-negative (HER2-n) one. Following the publication of the practice-changing “phase III ToGA” trial [4], trastuzumab (anti-HER2 drug)-containing regimen was licensed as the standard of care for HER2-p tumor both by the National Comprehensive Cancer Network (NCCN), European Society for Medical Oncology (ESMO), Japanese Gastric Cancer Association (JGCA) and Chinese Society of Clinical Oncology (CSCO) [5–8]. On the strength of the above guidelines, HER2 testing is strongly recommended in all patients with GC who are potential candidates for trastuzumab therapy [9]. Moreover, HER2 is thought to be a key driver of tumorigenesis in GC, and HER2-p status is suggested to be associated with the overall survival of the GC patient population, with poor outcomes might present in patients with HER2-p GC**.** [4]. Hence, discriminating HER2 positivity from HER2 negativity in patients with GC is essential for individualized management.

The immunohistochemistry (IHC)-based 4-tiered scoring system refined by Hofmann and colleagues [10] was recommended to decipher the HER2 status, with scores ranging from 0 (negative) to 3+ (positive). However, cases presenting with a score of 2+ are routinely classified to the equivocal group and should undergo an additional fluorescence in situ hybridization (FISH) or other in situ hybridization (ISH) procedures to further determine the classification [5, 8–10]. In other words, to identify the HER2 in all GC patients, testing pathologists often need to take two steps, performing IHC scoring first and followed by ISH methods in patients showing 2+ , which is undoubtedly technically inconvenient and cumbersome**.** This standard IHC/ISH technology, meanwhile, is initially based on relatively invasive biopsy or surgical specimens [8, 9]. Studies have been carried out to assess the potential relationship between HER2 expression and the noninvasive imaging tools, including PET/CT and conventional CT [11–13]; nevertheless, no deterministic conclusion and reliable model have been well established by far. In this way, a new initiative is needed to break the logjam.

Within the realm of nuclear medicine and medical imaging, research on radiomics in the field of oncology has grown exponentially over the last few years, revealing the potential of radiomics as a discipline to dramatically enhance medical care at various stages of the clinical pathway [14]. In terms of the molecular diagnostic of GC, two articles have been recently published to affirm the value of conventional CT radiomics for predicting the HER2 status [15, 16]. Albeit radiomics models have also been developed and tested, both studies presented certain methodological weaknesses, such as nonstandard grouping and informal quantitative validation. Therefore, herein we conducted this study to develop and validate a conventional CT-based diagnostic radiomics model so that a more evidence-based imaging biomarker (IB) to identify potential HER2-p candidates from HER2-n patients could be established. Moreover, external validation and evaluation were performed in GC patients with dual-energy CT (DECT) scans to explore the feasibility of generalizing the conventional CT-based radiomics model to DECT.

## Methods

### Study design and participants

The institutional review board of the First Affiliated Hospital of Zhengzhou University approved this retrospective, single-center, diagnostic study, and the requirements of patients' informed consent forms were waived. The study participants were recruited from the First Affiliated Hospital of Zhengzhou University from December 2011 to July 2020. The clinicopathological data and CT images of participants were derived from the collection of medical records and picture archiving and communication systems (PACS), respectively.

**Fig. 1:**
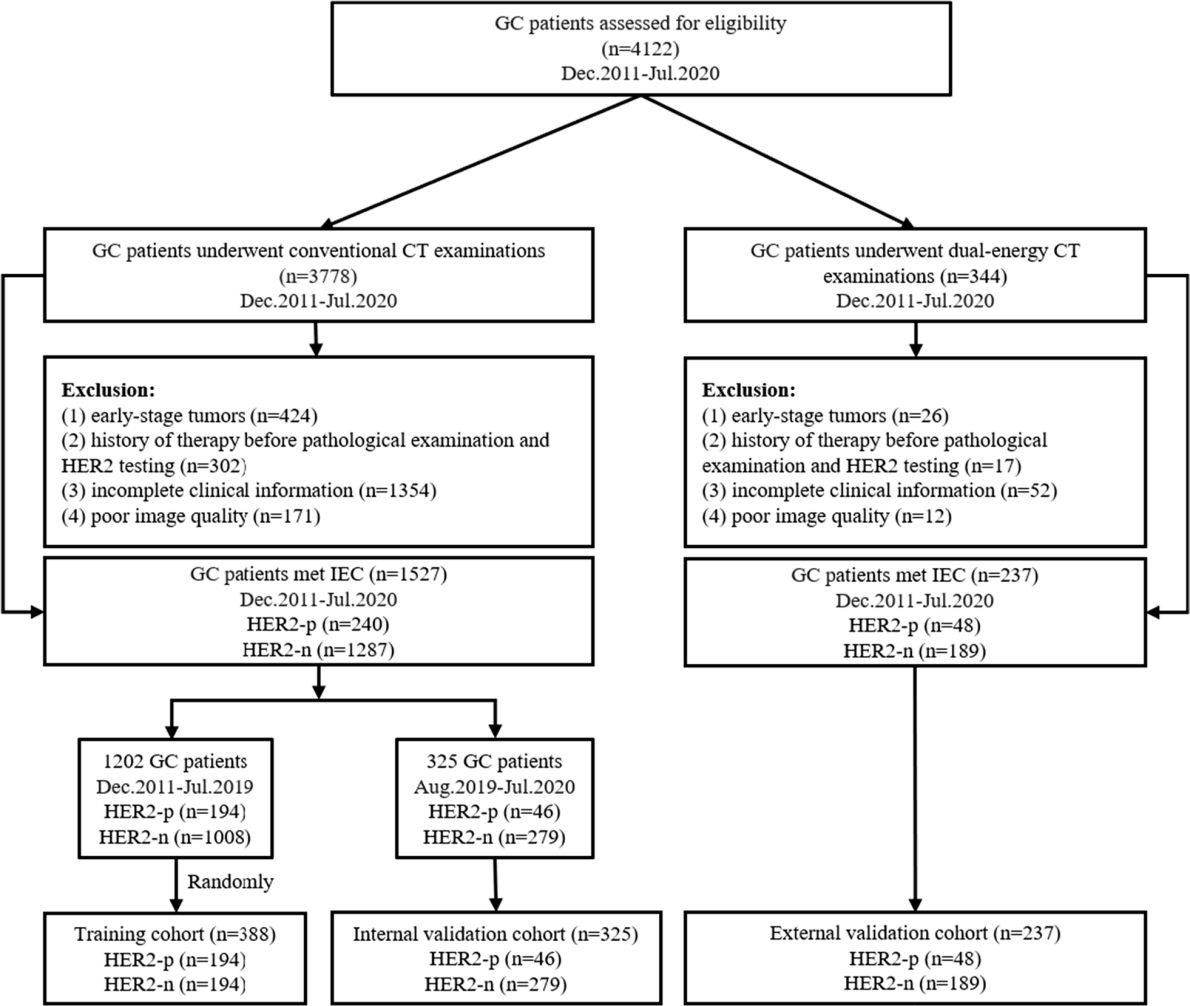
Study profile. Abbreviations: GC, gastric cancer; CT, computed tomography; IEC, inclusion/exclusion criteria; HER2-p, HER2-positive; HER2-n: HER2- negative

Details of the inclusion and exclusion criteria are presented in Additional file 1: Appendix E1. In terms of the sample size estimating, we balanced the efficiency of model building and the generalization ability of the results to the greatest extent, based on the explanations of the TRIPOD statement [17]. Finally, all selected subjects were assigned to three study cohorts according to the time order of diagnosis and CT protocol: a training cohort (December 2011 to July 2019, underwent conventional CT examinations; *n* = 388), an internal validation cohort (August 2019 to July 2020, underwent conventional CT examinations; *n* = 325) and an external validation cohort (December 2011 to July 2020, underwent DECT examinations; *n* = 237). The flow diagram of the patient population is displayed in Fig. 1, while the study-level characteristics are listed in Table 1.

**Table 1:** Baseline data for three gastric cancer (GC) study cohorts

Characteristics		Training cohort	Internal validation cohort	External validation cohort	*p* value
Line of care		Tertiary	Tertiary	Tertiary	–
Participants (*n*)		388	325	237	–
Age	> 60 y	200 (51.5)^a^	181 (55.7)^a^	112 (47.3)^a^	0.140
	≤ 60 y	188 (48.5)^a^	144 (44.3)^a^	125 (52.7)^a^	
Gender	Male	241 (62.1)^a^	227 (69.8)^a^	169 (71.3)^a^	0.025*
	Female	147 (37.9)^a^	98 (30.2)^a^	68 (28.7)^a^	
Type of tissue for confirmation	Biopsy tissue	164 (42.3)^a^	174 (53.5)^b^	100 (42.2)^a^	0.004*
	Surgical specimen	224 (57.7)^a^	151 (46.5)^b^	137 (57.8)^a^	
HER2 status	HER2-p	194 (50.0)^a^	46 (14.2)^b^	48 (20.3)^b^	< 0.001*
	HER2-n	194 (50.0)^a^	279 (85.8)^b^	189 (79.7)^b^	

Values are the number (percentage) unless otherwise indicated. HER2-p = HER2-positive, HER2-n = HER2-negative; * *p* < 0.05;

^**a,b**^pairwise comparison results. There was no difference between cohorts given consistent letters, while there was a statistically significant difference between cohorts given inconsistent letters

### HER2 status ascertainment

By using the tumor tissue in surgical or endoscopically biopsy specimens, pathologists performed IHC testing first, followed by ISH when the IHC result was 2+ (equivocal), to determine the HER2 classification. Ultimately, practitioners interpreted the HER2 test results as binary classification, namely HER2-p subtype (IHC 3+ or IHC 2+ plus FISH positivity) and HER2-n subtype (IHC 0 or IHC 1+ or IHC 2+ plus FISH negativity), according to the evidence-based guidelines [5, 9]. Additionally, to provide the reader insight into the differences in case-mix between study cohorts, the type of tissue confirmation for each patient was recorded and is summarized in Table 1.

### CT protocol

The details of the CT imaging protocol are available at Additional file 1: Appendix E2 and Table 2.

**Table 2:** CT imaging protocols

Parameters	Routine CT examinations	Dual-energy CT examinations
CT version	Phillips 256 iCT, Phillips Medical System, Netherlands; GE Discovery CT750 HD scanner or Revolution CT, GE Healthcare, USA	Spectral CT (GE Discovery CT750 HD scanner, GE Healthcare, USA)
CT tube voltage	120 kVp	Spectral imaging mode rapid switching between 80 and 140 kVp
CT tube current	120–550 mA	375 mA
CT rotation time	0.5 s	0.6 s
Contrast agent type	Omnipaque, GE Healthcare, USA	Omnipaque, GE Healthcare, USA
Contrast agent concentration	350 mgI/mL	350 mgI/mL
Contrast agent dosage	1.5 mL/kg body weight	1.5 mL/kg body weight
Contrast agent infused rate	3.0 mL/s	3.0 mL/s
Arterial phase interval time	30 s after injection of contrast agent	30 s after the injection of contrast agent
Venous phase interval time	70 s after injection of contrast agent	70 s after the injection of contrast agent
Field of view	500 × 500 mm	400 × 400 mm
Reconstruction/ image thickness	120 kVp; 0.625 mm or 1.25 mm or 5 mm	70 keV, 1.25 mm

### Clinical and laboratory potential candidate variables

The following clinical characteristics were collected for each patient: age, gender, tumor location (cardia vs non-cardia), size, thickness, characteristics of enhancement (mild or moderate vs obvious), Borrmann type (I + II vs III + IV), and clinical TNM staging (cTNM; I + II vs III + IV). Laboratory variables comprised carcinoembryonic antigen (CEA), carbohydrate antigen 125 (CA125), carbohydrate antigen 199 (CA199), carbohydrate antigen 724 (CA724), and carbohydrate antigen 153 (CA153), tested at the time of the primary diagnosis. The CT traits were independently evaluated by two gastrointestinal radiologists with 6 (reader 1) and 8 (reader 2) years of clinical experience in gastrointestinal CT imaging. The details are in Additional file 1: Appendix E3.

### Tumor segmentation and radiomics feature extraction

Venous phase CT images in a Digital Imaging and Communications in Medicine format were retrieved for tumor segmentation. Given the strong effect of spatial resolution on radiomic feature values, we conducted resampling to minimize the possible consequences of differences in resolution between CT scanners [18]. The multicentric images were resampled at a common voxel spacing of 1 mm × 1 mm × 1 mm using the linear interpolation technique. Following the pre-processing step, images were fed into the ITK-SNAP software (version 3.8.0; URL: http://www.itksnap.org) for two-dimensional region of interest (2D-ROI) segmentation on the maximal cross-sectional area of the tumor (Additional file 1: Appendix E4).

Radiomics features were extracted from original, local binary pattern (LBP) filtered, Laplacian of Gaussian (LoG) filtered, and wavelet-transformed images by using AK software (Artificial Intelligence Kit, Version V3.0.0.R, GE Healthcare) based on Python package PyRadiomics [19]. These features fell into six categories (Additional file 1: Appendix E4). Ultimately, a total of 1409 radiomics features were obtained per manually labeled 2D-ROI.

### Feature selection and radiomics signature building

The following processes were carried out to select the optimal subset of features in the training cohort. First, the intra-class correlation coefficient (ICC) was calculated based on the re-segmentation data to estimate intra- and interobserver reproducibility of features, and only those with ICCs value > 0.75 were reserved for subsequent analysis. Second, removed features with variance less than 1.0 and filled the missing values with the median. Z-score was used to standardize the features. Third, the standardized features were input into correlation analysis to retain non-redundant and irrelevant candidate feature sets with 0.9 as the cutoff value. Then, the importance of the features was ranked by the gradient boosted decision tree (GBDT), and features whose coefficients were greater than or equal to one-tenth of the maximum coefficient were retained. Finally, support vector machine (SVM) algorithm was implemented to construct the radiomics signature “Radscore” for each patient as a predictor of HER2 status in GC.

### Construction and quantitative evaluation of the radiomics model

In the training cohort, univariate analysis and multivariate logistic regression were applied to construct the radiomics model. First, differences in clinical-laboratory characteristics by HER2 status in the training cohort were compared using the univariate analysis, with a significance level for variable retention of 0.1. The multivariate logistic regression algorithm was subsequently performed to select the independent factors for HER2 status. Then, we integrated the radiomics signature with significant clinical-laboratory factors to develop a radiomics model using the same multivariable analysis, which was linearly fused with the significant predictors and corresponding regression coefficients. Finally, the generated radiomics model would be visualized as an easy-to-use nomogram for facilitating individual HER2 diagnosis in GCs.

To quantify any optimism and generalization for the diagnostic performance of the radiomics model, internal validation techniques and external validation methods were employed in separate data (internal validation cohort) and other participant data (external validation cohort). The discrimination and clinical usefulness of the nomogram were assessed with the area under curve (AUC) of receiver operating characteristic (ROC) curves and decision curve analysis (DCA), respectively. The 95% confidence intervals (CIs) of the AUCs were calculated, and Youden's index was also calculated to obtain the optimal cutoff value. Moreover, stratified analysis was conducted to evaluate the predictive efficacy and the certain universality of the model in different patient types based on all patients in the training, internal validation, and external validation cohorts. Delong test was called to compare the AUCs, so as to evaluate the stability of the established model.

### Statistical analysis

All calculations and statistical analyses were performed on SPSS software version 21.0 for Windows and R software package version 3.6.3 (URL: http://www.Rproject.org). A two-sided *p* value less than 0.05 was considered statistically significant. In univariate analyses, the Kolmogorov–Smirnov test was used to check the normality of continuous variables, and then differences were compared using either the Mann–Whitney *U* test or the independent *t* test, where appropriate, while the Chi-square test was utilized in the comparison of categorical variables. In performing the multivariate logistic regression analyses, we applied the likelihood ratio test based on the maximum partial likelihood estimates for identifying the key factors.

## Results

### Clinical characteristics

Of the 950 included GC patients in the three cohorts, 288 had a HER2-p status (244 had an IHC analysis score of 3+ , and 44 had an IHC analysis score of 2+ plus positive findings on FISH), and the remaining 662 had a HER2-n (620 had an IHC analysis score of 0/1+ and 42 had an IHC analysis score of 2+ plus negative findings on FISH). No significant difference was observed between the three cohorts in terms of the age at the first diagnosis (percentage of > 60 years, 51.5% vs 55.7% vs 47.3%, *p* = 0.140). However, there were differences in gender, the ratio of patients who had a surgical specimen analyzed, and the prevalence of HER2-p status to some extent (all *p* < 0.05; Table 1).

### Development of the radiomics signatures

Of 1409 radiomics features, 293 were shown to have good intra-and interobserver reproducibility, with ICCs > 0.75. After elimination by variance analysis, feature standardized and correlation analysis process, 43 non-redundant features were derived. GBDT importance ranking demonstrated that the maximum coefficient of radiomics features was 27.6603. Finally, 8 features with coefficients greater than 2.7660 were retained and used to establish the SVM-based Radscore (Additional file 1: Appendix E5).

### Construction and evaluation of the radiomics model

**Fig. 2:**
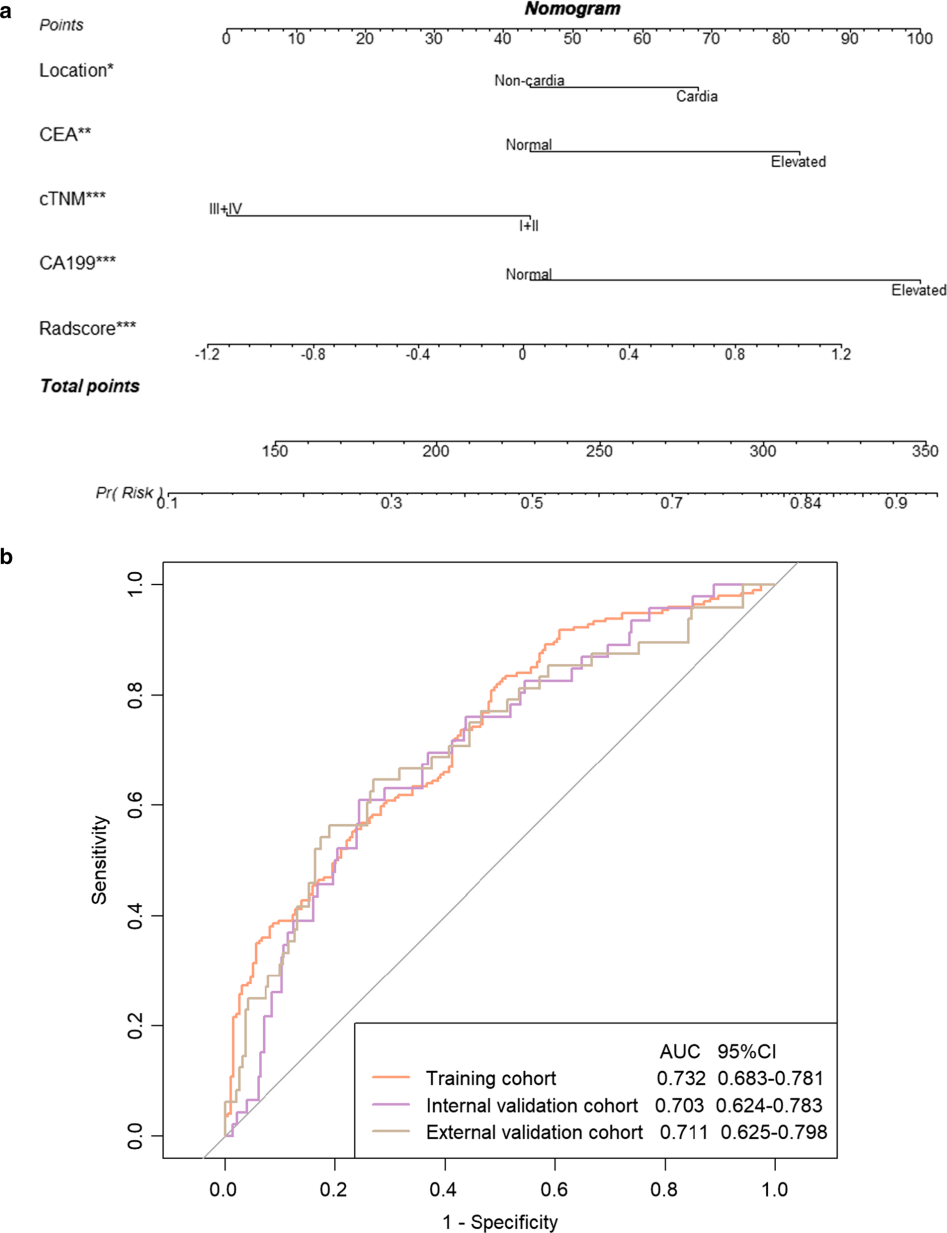
The visualized nomogram of the radiomics model (**a**) and receiver operating characteristic (ROC) curves in the training, internal validation, and external validation cohorts (**b**). **a** From each predictor, draw a vertical line up through the “Point” scale (the top line) to get the point and then sum all points from each predictor. Next, find the sum value in the “Total Points” scale and draw a vertical line through the “Pr (risk)” scale (the bottom line) to get the final predicted probability for HER2 positivity. CEA = carcinoembryonic antigen; cTNM = clinical TNM staging; CA199 = carbohydrate antigen 199; Radscore = radiomics signature; number of asterisks (*) listed after each variable denotes inverse association with *p* value for that variable. Single asterisk indicates *p* = 0.044, double asterisk indicates *p* = 0.003, and triple asterisk indicates *p* < 0.001. **b** AUC = area under the receiver operating characteristic curve; CI = confidence interval

**Fig. 3:**
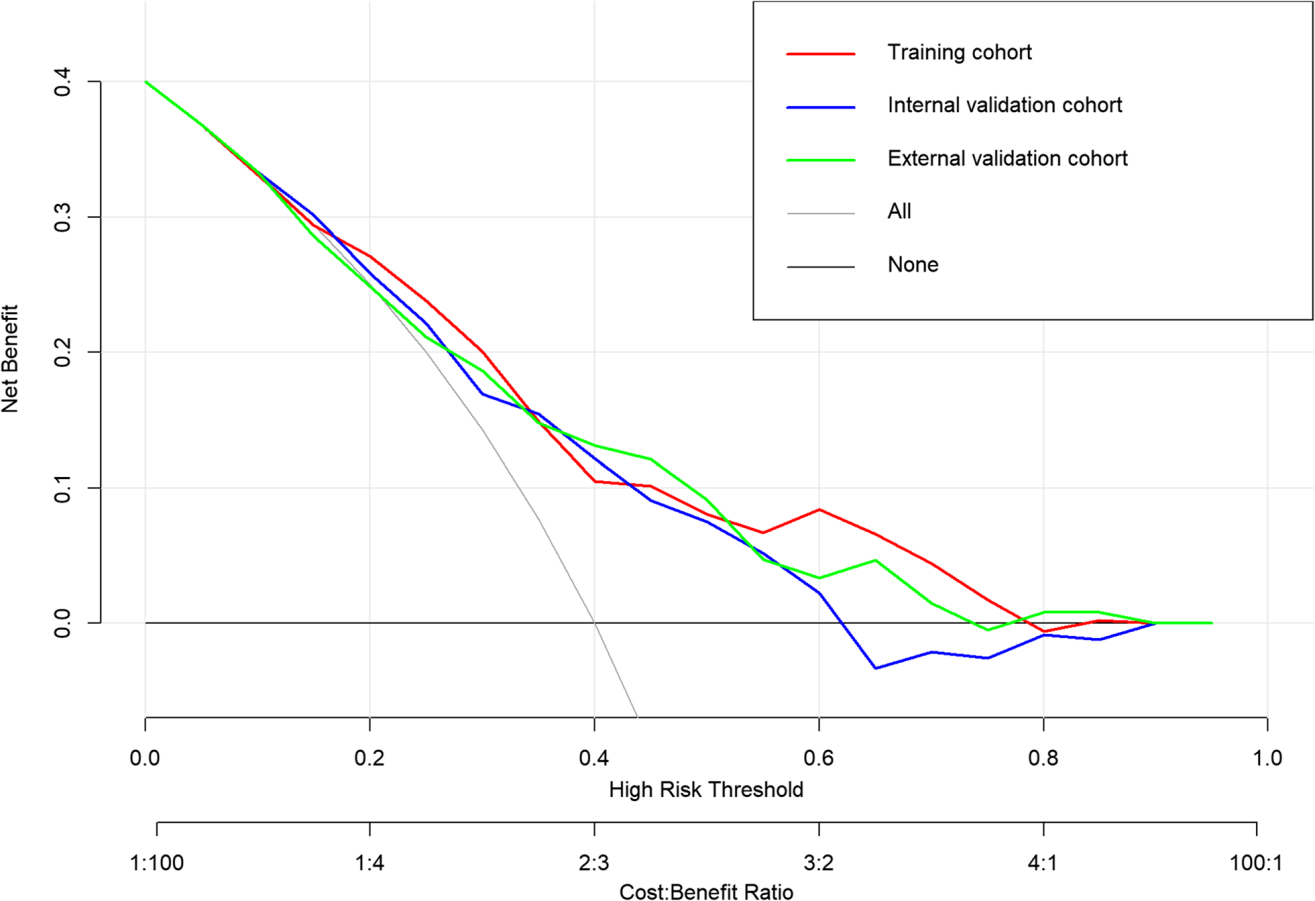
Decision curves of the radiomics model in the training, internal validation, and external validation cohorts. The X-axis represents the threshold probability and the Y-axis is the net benefit. The black line “NONE” indicates that no lesions are assumed to be HER2-positive, and the gray line “ALL” indicates that all lesions are assumed to be HER2-positive. The model with higher clinical usefulness means it is further away from both the black and gray lines

In the training cohort, univariate analysis and multivariate logistic regression of clinical-laboratory characteristics and Radscore revealed that tumor location, cTNM staging, CEA, CA199, and Radscore were significant predictors for HER2 status of GCs (Table 3). And patients with HER2-p tumors showed a predominance of the cardiac anatomic subsite, a higher stage I/II ratio, and higher CEA and CA199 elevation ratios. Therefore, they were fused as a radiomic model and visualized as a radiomics nomogram (Fig. 2A). The nomogram yielded diagnostic ability of HER2-p status in the training, internal validation, and external validation cohorts, as AUCs of 0.732 (95%CI 0.683–0.781) and 0.703 (95%CI 0.624–0.783), and 0.711 (95%CI 0.625–0.798), respectively (Fig. 2B). The optimal cut-off value corresponding to the maximized Youden’s index (0.3250) was selected as 0.4132. Delong test showed no statistical differences between the AUCs in the three cohorts (*p* = 0.546 (training cohort *vs* internal validation cohort), 0.678 (training cohort *vs* external validation cohort), and 0.897 (internal validation cohort *vs* external validation cohort)), indicating that the conventional CT radiomics-based model was robust and may be generalized to the GC populations that underwent DECT scans. DCA suggested that the radiomics model might provide a higher net benefit than simple “all HER2-targeted therapy” (all HER2-p) or “none HER2- targeted therapy” (none HER2-p) strategies for patients in all three study cohorts, with threshold probabilities ranging from 10 to 80%, from 20 to 65%, and from 10 to 75%, respectively (Fig. 3).

**Table 3:** Univariate analysis and multivariate logistic regression analysis of the clinical-laboratory characteristics and Radscore in training cohort

Parameters		Univariate analysis		Multivariate analysis	
		Statistic	*p* value	OR (95% CI)	*p* value
Age		0.421	0.674		
Gender	Male/Female	4.830	0.028^*^	1.4 (0.9–2.2)	0.122
Location	Cardia/Non-cardia	12.629	< 0.001^*^	1.6 (1.0–2.6) ^**a**^	0.044^**^
cTNM staging	I + II/III + IV	6.292	0.012^*^	0.4 (0.3–0.7) ^**a**^	< 0.001^**^
Borrmann type	I + II/III + IV	0.538	0.463		
Size		− 1.494	0.136		
Thickness		0.990	0.323		
Characteristics of enhancement	Mild or moderate/Obvious	0.513	0.474		
CEA	Normal/Elevated	14.458	< 0.001^*^	2.1 (1.3–3.6)^**a**^	0.003^**^
CA125	Normal/Elevated	2.456	0.117		
CA199	Normal/Elevated	15.559	< 0.001^*^	3.0 (1.7–5.4)^**a**^	< 0.001^**^
CA724	Normal/Elevated	4.108	0.043^*^	1.5 (0.8–2.7)	0.172
CA153	Normal/Elevated	3.744	0.053^*^	1.8 (0.6–5.1)	0.262
Radscore		5.333	< 0.001^*^	2.1 (1.5–3.0)^**a**^	< 0.001^**^

Note—OR = odds ratio; CI = confidence interval; cTNM staging = clinical TNM staging; CEA = carcinoembryonic antigen; CA125 = carbohydrate antigen 125; CA199 = carbohydrate antigen 199; CA724 = carbohydrate antigen 724; CA153 = carbohydrate antigen 153; Radscore = radiomics signature; * *p* < 0.1; ** *p* < 0.05;

^**a**^Adjusted OR (95% CI)

**Fig. 4:**
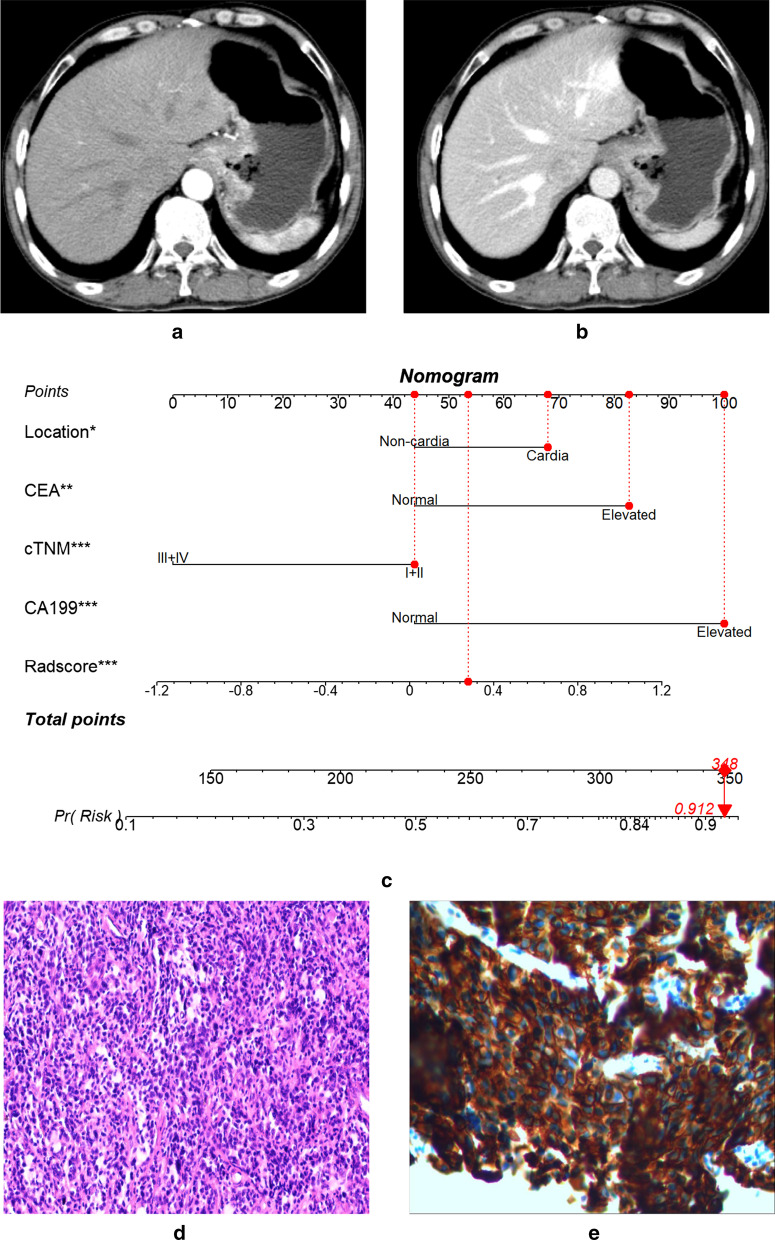
63-year-old man with gastric cancer (GC). Arterial phase (**a**) and venous phase CT image (**b**) showed that the lesion was located in the cardia of the stomach and had a venous phase CT-based Radscore of 0.277840926. According to the CT images and other data, the patient was diagnosed as stage II in terms of clinical TNM (cTNM) staging. Laboratory examination at initial diagnosis showed that the patient had elevated carcinoembryonic antigen (CEA) and carbohydrate antigen 199 (CA199). Nomogram (**c**) showed that when points for individual predictors were added based on the “Points” scale in the top row, the total points were 348, and the probability of the patient having HER2-positive GC was 91.2%. Histopathological HE staining (**d**) and immunohistochemistry (IHC, **e**) confirmed an IHC 3 + gastric adenocarcinoma, namely HER2-positive GC

To test the generalization ability of the established radiomics model, we performed stratified analysis on the subgroups of gender, age, tumor location, IHC results, and type of tissue for confirmation. We used the ROC curves and AUCs to evaluate the model performance in these subpopulations. As shown in Additional file 1: Fig. S1 and Table 4, the results substantiated that the performance of our radiomics model was not influenced by these factors (Delong test, all *p* > 0.05), suggesting its generality in different types of patients. In addition, two examples of applying the radiomics nomogram to predict the HER2 positivity in patients with GCs are shown in Fig. 4 and Additional file 1: Fig. S2.

**Table 4:** The performance of the radiomic nomogram in stratified analysis

Patient types		AUC	95%CI		*p* value
			Lower	Upper	
Gender	Male	0.710	0.668	0.752	0.802
	Female	0.720	0.652	0.789	
Age	> 60 y	0.710	0.662	0.759	0.730
	≤ 60 y	0.723	0.671	0.775	
Location	Cardia	0.683	0.633	0.734	0.556
	Non-cardia	0.706	0.650	0.762	
IHC results	IHC 2+	0.658	0.539	0.776	0.291
	IHC others	0.725	0.687	0.763	
Type of tissue for confirmation	Biopsy tissue	0.699	0.645	0.754	0.319
	Surgical specimen	0.736	0.690	0.782	

AUC = area under the receiver operating characteristic curve; CI = confidence interval; IHC = immunohistochemistry

## Discussion

Subclassification of GC as HER2-p or HER2-n subtype has implications for clinical therapy and prognosis [4, 5, 8]. Nevertheless, the officially recommended, currently used HER2 testing systems are labor-intensive and relatively invasive. As a result, diagnostic researches based on non-invasive imaging techniques have been rapidly expanding [11–13, 15, 16]. However, these studies lack standardized design, external validation analysis, or completed and accurate reporting. Consequently, we conducted this large-scale experiment to integrate a CT radiomics-based prediction model for identifying HER2-p status in GCs. Subsequently, we verified the performance of the achieved model in independent cohorts of patients. This study displayed that the established model yielded stable predictive ability in the training and validation cohorts, implying the presented nomogram is of certain clinical application potential in quantifying an individual’s risk of HER2 overexpression-positive status.

Concerning the focused imaging modality in this article, CT was chosen due to its “first-line” nature in assessing GCs. It is worth mentioning that two studies based on CT radiomics have been put into effect to predict the HER2 status of GCs [15, 16]. The results suggested that CT-based radiomics offers great promise to aid in the preoperative evaluation of HER2 expression in GC patients. Nevertheless, the most vulnerable point of these works lain in the experimental design. According to the guidelines [5, 9], both biopsy and postoperative specimens can be used for HER2 detection, and IHC plus ISH technology are required to stratify patients, so studies focused on either alone (postoperative specimens-based ISH analysis or IHC test as golden standard) could result in bias. What’s more, to date, no clear benefit of trastuzumab in patients with early-stage GC has been supported by high-quality pieces of evidence [9]. Therefore, the inclusion of patients with T1 staged tumors in HER2 prediction practice is insufficiently evidenced. In the current study, we carefully dealt with the above issues, and the good news was that the model we achieved had acceptable diagnostic efficiency, diagnostic stability and good generalization ability.

In our study, the overall HER2-p rate of patients initially meeting the inclusion and exclusion criteria was 16.32% (288/1764). While inter-setting variations existed, the incidence estimates were all within the previously reported prevalence ranges of 12% to 23% [5]. Noteworthy, recent evidence suggested that the overall incidence rates of HER2 overexpression in metastatic gastroesophageal cancer increased over time, but the number of negative GC patients was still overwhelmingly dominant [20]. Back to this study, our primary objective required the maximum number of patients to maximize the predictive model's power and generalization ability. However, the prevalence of our target condition (HER2-p) was too low, and the cost of measuring predictors was too high to warrant model construction efficiency. Therefore, we used all HER2-p cases but a random sample of HER2-n patients in the training cohort, and all available subjects correspond to real-world proportions in the validation cohorts. At present, such a sampling design is attractive in situations like ours and necessary for use in some diagnostic prediction modeling studies to obtain unbiased absolute probabilities [17, 21, 22].

Radiomics and prediction-model establishing studies pursue validation techniques, especially prefer external validation [14, 17]. As suggested, we performed internal validation and included other participant datasets (external validation cohort) to assess model performance. Most notably, the external validation cohort we specifically set up was a separate data cohort in which patients with 70 keV virtual monochromatic images (VMIs) were enrolled. Recently, the DECT platform is gaining importance in cancer assessments [23, 24], raising whether the conventional CT-based model was also applicable to DECT-derived images, namely the higher level of generalization. Prior analyses have proven that VMIs with an energy level at approximately 70 keV were comparable to conventional 120 kVp CT images [25–27]. In view of the statements identified above, we hypothesized that 120 kVp image-based model could generalize to 70 keV VMIs. Our experimental results showed that the radiomics model exhibited good performance in discriminating HER2 status in the training cohort and elicited similar discriminatory capability on validation cohorts containing the DECT one. In consideration of the observations, we speculated that the radiomics features yielded from these two types of images were correlated, whereas we have not retrieved any literature aiming at the relationship between the radiomics data of the two yet. Thus, the certainty of our conjectures and the underlying basis of this preliminary finding may require further investigation.

In our statistical analyses, a significantly higher CEA and CA199 positivity ratio in HER2-p group were elucidated. Although past studies have found the trend of a significant association between CEA and HER2 expression [15, 28], the rationality and the positive correlation between CA199 and HER2 have not been confirmed. As previously reported, CEA, CA199, and HER2 had more positivity frequency for cancers at the upper third of the stomach [3, 4, 29, 30]. Accordingly, the tumor marker-dependent increase in HER2-p rates in our study may result from the link between them and tumor location. Owing to the sparse knowledge on the relationship of CEA and CA199 to HER2 overexpression, we performed further analyses with all study subjects and likewise discovered that both were significant indicators for HER2 status. Furthermore, consistent with the above-mentioned reports [3, 4], our data confirmed the anatomic subsites predominance of HER2 positivity. Similarly, the study demonstrated that the incidence of HER2 positivity was significantly higher in male patients, as Sheng et al. have explicitly presented [30]. However, multivariate analysis showed that gender was not an independent risk factor for predicting HER2 status of GC. These indicated that the easy-to-obtain clinical-laboratory variables have a latent force to be biomarkers for selecting HER2-p patients. Care providers should, therefore, routinely test the serum tumor markers.

This diagnostic investigation with a wealth of data also identified a significant association between image-based signature and HER2 positivity. Adding this CT-associated radiomics signature to the independent clinical-laboratory predictors resulted in a radiomics model with better discriminative power. One suggested explanation is that radiomics can capture and quantify the intratumor heterogeneity in medical images, where information related to HER2 expression is transformed into quantitative features that are further integrated into the imaging phenotypes of HER2 status [14, 31]. In addition, stratified analyses indicated that the HER2-specific radiomics nomogram was generalizable across different patient classes. Although guidelines recommend further ISH testing for HER2 IHC 2+ cases [5, 9], the high expense hinders the optimal clinical implementation. Hence, our predictive model has the potential to expand the patient population receiving anti-HER2 treatment while reducing medical expenditure. More importantly, to facilitate the usage, re-validation, and continuous updating of our model, the report adheres to the TRIPOD statement for prediction model studies [17]. In these respects, to our knowledge, previous HER2-related model studies have not performed very well and are therefore of insufficient overall quality. Finally, if the identified model is persistently optimized and then fortunately applied to the clinic by crossing ‘translational gaps’ [32], a one-step identification of HER2 status will be achieved, that is, a truly ‘digital biopsy’.

There were several limitations to this study. First, since we used the retrospective datasets to derivate and validate the model, the current findings need to be further verified in a prospective cohort. Second, initially, we determined the sample size according to practical considerations, but excluding patients with missing clinical-laboratory data in the subsequent modeling process may lead to selection bias. Third, we performed one-slice 2D rather than whole-volume 3D analyses. Although one previous study recommended using time-saving 2D delineation in GC radiomics-based research [33], there's no denying that multi-slice 3D descriptors carry more information than 2D annotations. Therefore, future volumetric analyses deserve more attention. Finally, in this preliminary study, we only included venous phase CT images for feature extraction, additional studies using multi-phase 120-kVp CT images and VMIs at whole energy levels are desirable.

## Conclusion

In this study, we built and validated a conventional CT-based radiomics model that achieves a good distinction capability for decoding the HER2 positivity of patients with GCs, and has the potential to generalize to DECT. The fused HER2-predicting nomogram could function as an IB with the potential to streamline the clinical workflows and aid healthcare providers in a preliminary screening of underlying candidates who might derive benefit from HER2-directed therapy. Continually revisiting the precision of the newly established IB and ameliorating the emerged nomogram are warranted.

## Supplementary Information

**Additional file 1.** Supplementary material.

## Data Availability

The datasets used and/or analyzed during the current study are available from the corresponding author on reasonable request.

## Abbreviations

AUC: Area under curve
DCA: Decision curve analysis
DECT: Dual-energy CT
FISH: Fluorescence in situ hybridization
GBDT: Gradient boosted decision tree
GC: Gastric cancer
HER2: Human epidermal growth factor receptor 2
IB: Imaging biomarker
ICC: Intra-class correlation coefficient
IHC: Immunohistochemistry
PACS: Picture archiving and communication systems
ROC: Receiver operating characteristic
SVM: Support vector machine
TRIPOD: Transparent reporting of a multivariable prediction model for individual prognosis or diagnosis
