# Supplementary material for: Development and external validation of a radiomics model for assessment of HER2 positivity in men and women presenting with gastric cancer
Source: Insights Imaging. 2023 Feb 1;14:20. doi: 10.1186/s13244-022-01361-x (PMC9889592; doi:10.1186/s13244-022-01361-x)
Supplement: Supplementary file 1 — Additional file 1. Supplementary material. [file 13244_2022_1361_MOESM1_ESM.pdf]

ELECTRONIC SUPPLEMENTARY MATERIAL

Development and external validation of a radiomics model for assessment of HER2 positivity in men and women presenting with gastric cancer

This supplementary material includes:

Appendix E1: Study design and participants

Appendix E2: CT protocol

Appendix E3: Clinical and laboratory potential candidate variables

Appendix E4: Tumor Segmentation and Radiomics Feature Extraction

Appendix E5: Development of the radiomics signatures

References for Supplementary Material

Supplementary Figures

Methods

Appendix E1: Study design and participants

Eligible patients were aged at least 18 years, presenting with histologically confirmed adenocarcinoma of the stomach, had tested the HER2 status, and underwent abdominal contrast-enhanced CT examinations during diagnosis. Major exclusion criteria included patients with early-stage tumors diagnosed either by postoperative pathological analysis or the dedicated GC team through screening patients' images and clinicopathologic data, history of radiotherapy, chemotherapy, and targeted therapy prior to the pathological examination and HER2 testing, incomplete clinical information, poor image quality.

Appendix E2: CT protocol

After making the necessary pre-examination preparations, all patients underwent abdominal unenhanced and contrast-enhanced CT during breath-hold with the head-first supine position. The major preparations included: (1) fasted overnight to empty the gastrointestinal tract; (2) 15-20 min prior to the CT scan, intramuscularly injected 20 mg scopolamine (Hangzhou Minsheng Pharmaceutical Group Co., Ltd. Specifications: 10 mg/ml) to minimize gastrointestinal peristalsis; (3) drank 800-1000 ml warm water 5 min before scanning to dilate the stomach. The CT imaging protocol are listed in Table 2. The CT images were reconstructed using a standard kernel, with reconstruction section thicknesses ranging from 0.625 mm to 5mm.

Appendix E3: Clinical and laboratory potential candidate variables

In the qualitative interpretation, when any disagreement existed, an expert-level radiologist was consulted for a final diagnosis. As for the quantitative analyses, the mean values were calculated and applied. Before the assessment, all observers were informed that the study population had GCs, but were blinded to other pertinent clinical information and histopathological data. Tumor size and thickness were defined as the maximal diameter and the longest diameter perpendicular to the long axis on the largest cross-section, respectively. Enhancement characteristics were determined by the difference between the CT values of the tumor in dynamic enhanced and unenhanced CT images, with the difference of > 40 HU as an obvious enhancement, and ≤ 40 HU as a mild or moderate enhancement. cTNM staging was determined according to the criteria proposed in the journal of European Radiology and the 8th edition AJCC/UICC clinical staging system [1, 2].

Appendix E4: Tumor segmentation and radiomics feature extraction

The ROIs were manually delineated to outline the visible tumor, carefully maintaining approximately 2-3 mm from the margin to reduce bias caused by the partial volume effect. All images were segmented by reader 1 first, then to evaluate intra- and interobserver reproducibility, reader 1 and reader 2 repeated the segmentation one month later with 50 randomly selected patients.

These extracted features fell into six categories: (1) shape-based features (n=14); (2) first-order statistics features (n=18); (3) textural features extracted from the following matrices (n=75): gray level cooccurrence matrix (GLCM, n=24), gray level run length matrix (GLRLM, n=16), gray level size zone matrix (GLSZM, n=16), neighboring grey tone difference matrix (NGTDM, n=5) and gray level dependence matrix (GLDM, n=14); (4) LBP filtered images-based features (n=279); (5) LoG filtered first-order and textural features with sigma of 1.0, 3.0 mm and 5.0 mm (n=279) and (6)

wavelet-transformed first-order and textural features in frequency channels LLL, HHL, HHH, HLH, LLH, LHH, LHL and HLL (n=744). Detailed feature descriptions and mathematic formulas can be easily found on the publicly available library PyRadiomics (URL: http://pyradiomics.readthedocs.io/en/latest/features.html).

Results

Appendix E5: Development of the radiomics signatures

The features included in Radscore construction were original_firstorder_Mean, wavelet.HHL_firstorder_Variance, log.sigma.5.0.mm.3D_gldm_GrayLevelVariance, lbp.3D.k_ngtdm_Busyness, wavelet.LHH_glszm_GrayLevelNonUniformity, lbp.3D.k_glrlm_RunLengthNonUniformity, wavelet.LHH_glrlm_RunLengthNonUniformity, and log.sigma.5.0.mm.3D_gldm_LargeDependenceEmphasis, with corresponding importance coefficients of 27.6603, 17.9662, 14.8314, 14.2874, 11.0873, 6.4603, 4.1550, and 3.5518, respectively.

References for Supplementary Material

1. Kim JW, Shin SS, Heo SH et al (2012) Diagnostic performance of 64-section CT using CT gastrography in preoperative T staging of gastric cancer according to 7th edition of AJCC cancer staging manual. Eur Radiol 22(3):654-662
2. Amin MB, Edge SB, FL G (2017) AJCC Cancer StagingManual. 8th ed [M]. New York: Springer.

Results

Supplementary Figures

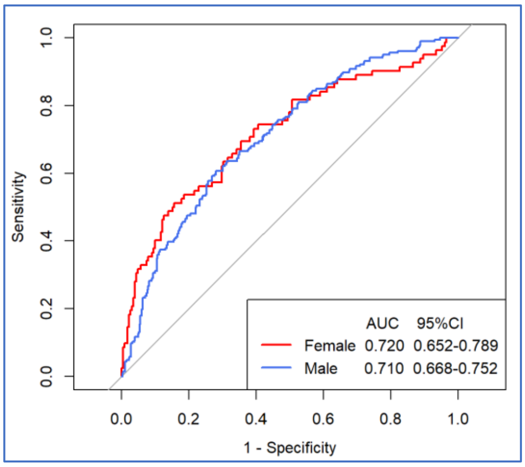

a

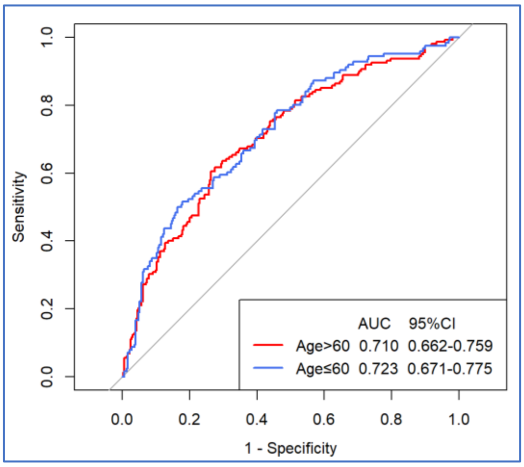

b

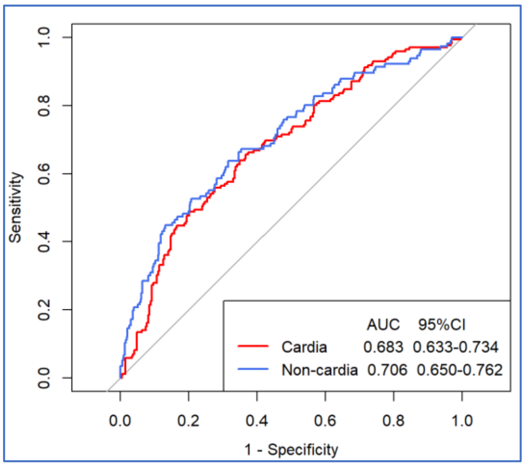

c

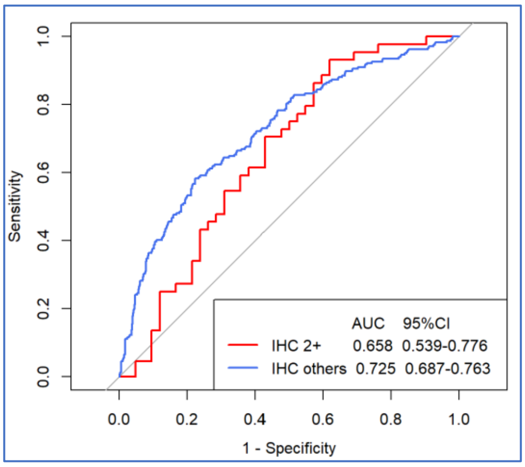

d

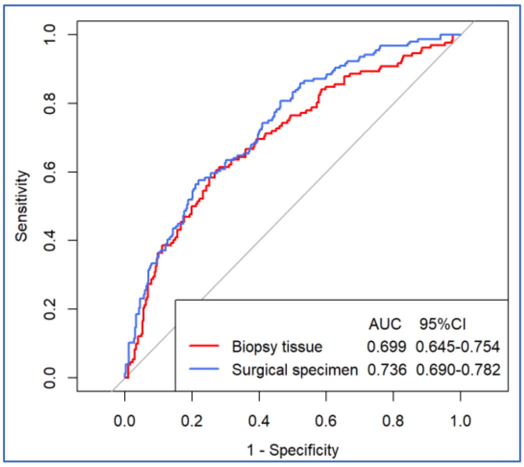

e

Fig. S1: Stratified analysis of the performance of radiomics model by gender (**a**), age (**b**), tumor location (**c**), IHC results (**d**), and type of tissue for confirmation (**e**). AUC = area under the receiver operating characteristic curve; CI = confidence interval; IHC = immunohistochemistry.

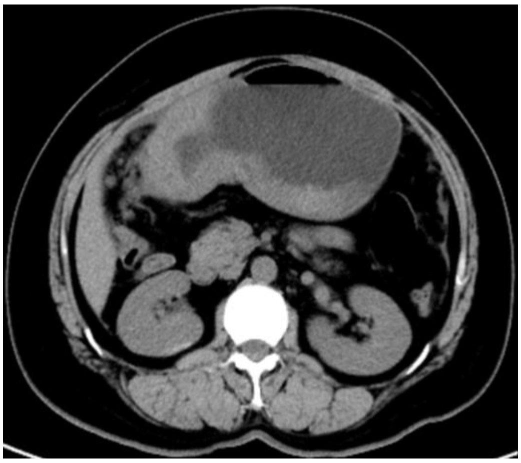

a

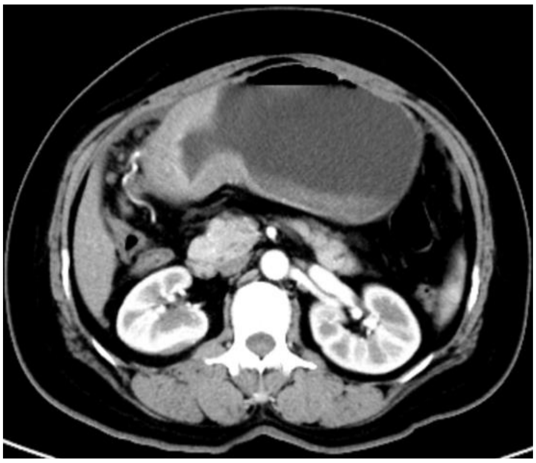

b

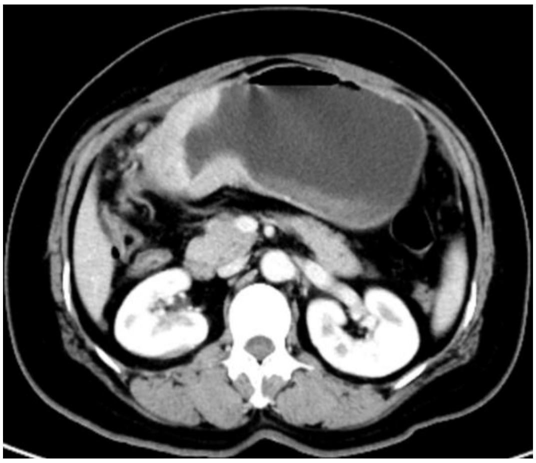

c

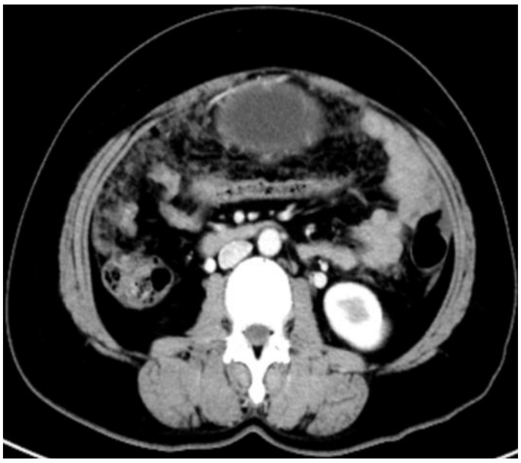

d

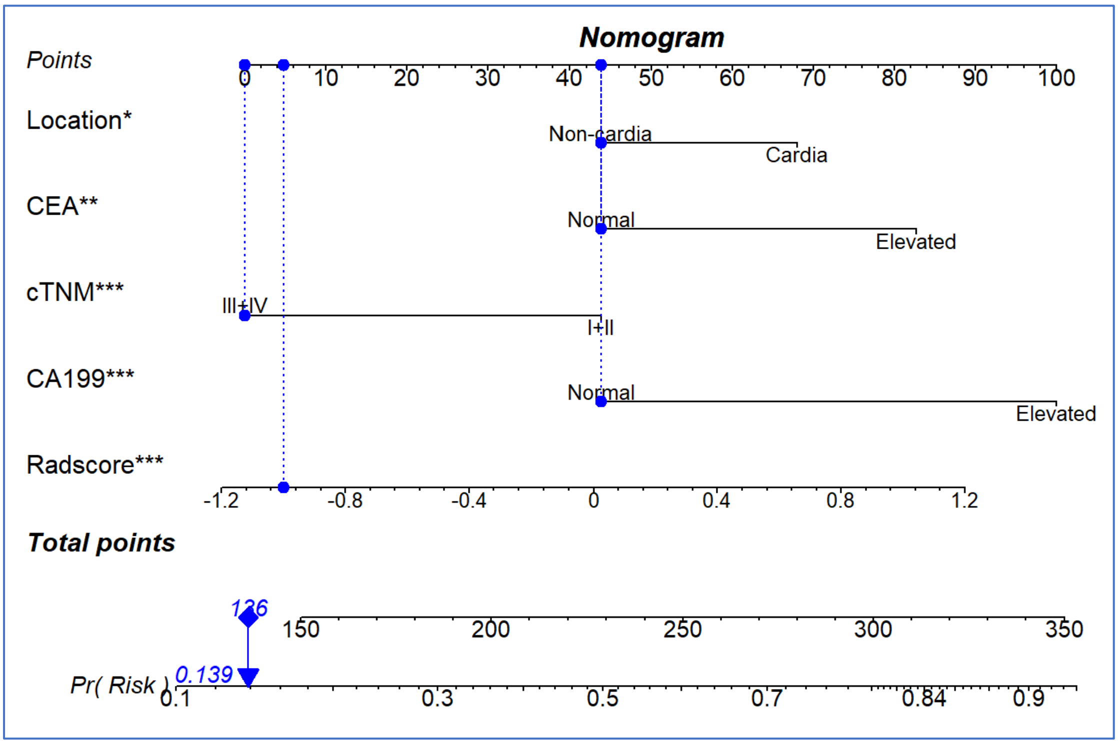

Nomogram
Points
0 10 20 30 40 50 60 70 80 90 100
Location*
Non-cardia
Cardia
CEA**
Normal
Elevated
cTNM***
III+IV
I+II
CA199***
Normal
Elevated
Radscore***
-1.2 -0.8 -0.4 0 0.4 0.8 1.2
Total points
126
150 200 250 300 350
Pr(Risk)
0.139
0.1 0.3 0.5 0.7 0.84 0.9

e

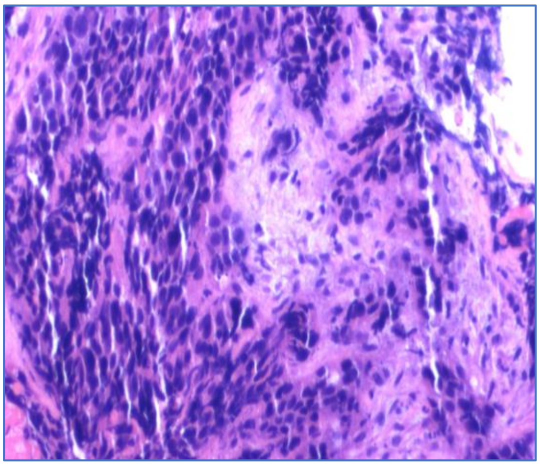

f

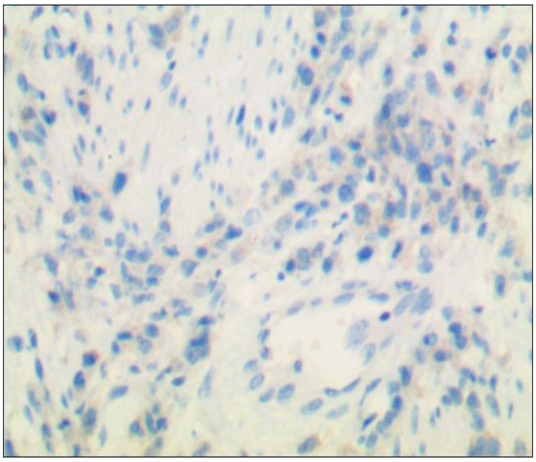

g

Fig. S2: 42-year-old woman with gastric cancer (GC). Pretreatment unenhanced (**a**) and contrasted enhanced CT images (**b-c**) showed that the lesion was located in the antrum of the stomach, accompanying with obvious peritoneum thickening (**d**). According to the CT images and other data, the patient was diagnosed as stage IV in terms of clinical TNM (cTNM) staging and had a venous phase CT-based Radscore of -1.000035812. Laboratory examination at initial diagnosis showed that the patient had normal carcinoembryonic antigen (CEA) and carbohydrate antigen 199 (CA199). Nomogram (**e**) showed that when points for individual predictors were added, the total points were 126, and the probability of the patient having HER2-positive GC was 13.9%. Histopathological HE staining (**f**) and immunohistochemistry (IHC, **g**) revealed the patient had IHC 0 gastric adenocarcinoma, namely HER2-negative GC.
